# Physical and Physiological Match-Play Demands and Player Characteristics in Futsal: A Systematic Review

**DOI:** 10.3389/fpsyg.2020.569897

**Published:** 2020-11-06

**Authors:** Konstantinos Spyrou, Tomás T. Freitas, Elena Marín-Cascales, Pedro E. Alcaraz

**Affiliations:** ^1^UCAM Research Center for High Performance Sport, Catholic University of Murcia, Murcia, Spain; ^2^NAR – Nucleus of High Performance in Sport, São Paulo, Brazil; ^3^Faculty of Sport Sciences, Catholic University of Murcia, Murcia, Spain

**Keywords:** five-a-side soccer, game-analysis, performance, physical capacities, team-sports

## Abstract

Futsal, also known as five-a-side indoor soccer, is a team-sport that is becoming increasingly popular. In fact, the number of futsal-related investigations is growing in recent years. This review aimed to summarize the scientific literature addressing the match-play demands from the following four dimensions: time-motion/external load analysis and physiological, neuromuscular, and biochemical responses to competition. Additionally, it aimed to describe the anthropometric, physiological, and neuromuscular characteristics of elite and sub-elite male futsal players, contemplating the differences between competition levels. The literature indicates that elite futsal players cover greater total distance with higher intensities and perform a greater number of sprints during match-play when compared to sub-elite players. The physiological demands during competition are high (average intensity of ≥85% maximal heart rate and ~80% maximum oxygen uptake [VO_2max_]), with decrements between the two halves. Research suggests that neuromuscular function decreased and hormonal responses increased up to 24 h after the match. Considering anthropometric characteristics, players present low percentage of body fat, which seems commonplace among athletes from different on-court positions and competition levels. Elite players display greater values and at VO_2max_ with respect to sub-elite competitors. Little is known regarding elite and sub-elite futsal players' neuromuscular abilities (strength, jumping, sprinting, and change of direction [COD]). However, it appears that elite players present better sprinting abilities compared to lower-level athletes. Futsal players aiming to compete at the highest level should focus on developing maximal speed, lower-body power and strength, aerobic capacity, and lean muscle mass.

## Introduction

Futsal, also known as five-a-side indoor soccer, is a team-sport officially authorized by FIFA and is becoming increasingly popular all over the world. It is characterized as a high-intensity intermittent sport that imposes high physical, technical, tactical, and psychological demands on players (Barbero-Alvarez et al., 2008). The game is played five-a-side (i.e., four on-court players and one goalkeeper), in a 40 × 20 m court, with a 3 × 2 m goal post and an unlimited number of substitutions. The maximum number of players in a squad for a match is 14 (a maximum of 9 substitutes per team) (FIFA, 2020). A futsal match consists of two halves of 20 min separated by a 10 min break. Given that the game-clock is stopped for some events (i.e., ball out of the court, faults, corners), a competitive match may last between 75 and 90 min (Álvarez et al., 2002). During match-play, teams can request one timeout (1 min) in each half.

Of note, the number of futsal-related investigations is growing in recent years. Several studies have described competition demands (Dogramaci and Watsford, 2006; Barbero-Alvarez et al., 2008; Castagna et al., 2009; Dogramaci et al., 2011; Makaje et al., 2012; Bueno et al., 2014; Caetano et al., 2015; Milioni et al., 2016; Milanez et al., 2020; Ohmuro et al., 2020; Ribeiro et al., 2020; Yiannaki et al., 2020) by reporting the physiological (Barbero-Alvarez et al., 2008; Castagna et al., 2009; Rodrigues et al., 2011; Makaje et al., 2012; Charlot et al., 2016; Milioni et al., 2016; Bekris et al., 2020; Yiannaki et al., 2020), neuromuscular (Caetano et al., 2015; Milioni et al., 2016; Milanez et al., 2020; Ribeiro et al., 2020), or biochemical responses (Moreira et al., 2011; de Moura et al., 2013; Bekris et al., 2020) following a competitive match. In addition, different authors have shown particular interest in describing the characteristics of futsal players such as anthropometrics (Baroni and Leal Junior, 2010; Gomes et al., 2011; Jovanovic et al., 2011; Garrido-Chamorro et al., 2012; de Moura et al., 2013; Pedro et al., 2013; Ramos-Campo et al., 2014; Galy et al., 2015; Nikolaidis et al., 2019; López-Fernández et al., 2020) and physiological (Barbero-Alvarez et al., 2009; Gorostiaga et al., 2009; Baroni and Leal Junior, 2010; Castagna and Barbero-Alvarez, 2010; Milanez et al., 2011; Makaje et al., 2012; Boullosa et al., 2013; Oliveira et al., 2013; Pedro et al., 2013; Cuadrado-Peñafiel et al., 2014; Miloski et al., 2014; Soares-Caldeira et al., 2014; De Freitas et al., 2015, 2019; Galy et al., 2015; Garcia-Tabar et al., 2015; Charlot et al., 2016; Floriano et al., 2016; Nakamura et al., 2016, 2018; Naser and Ali, 2016; Barbieri et al., 2017; Barcelos et al., 2017; Valladares-Rodriguez et al., 2017; Nogueira et al., 2018; Zarebska et al., 2018, 2019; Farhani et al., 2019; Nikolaidis et al., 2019; Teixeira et al., 2019; Wlodarczyk et al., 2019, 2020; Bekris et al., 2020) and neuromuscular qualities (Gorostiaga et al., 2009; Gomes et al., 2011; Cuadrado-Peñafiel et al., 2014; Soares-Caldeira et al., 2014; Galy et al., 2015; Charlot et al., 2016; Miloski et al., 2016; Nakamura et al., 2016; Naser and Ali, 2016; Vieira et al., 2016; De Lira et al., 2017; Loturco et al., 2018, 2020; Nogueira et al., 2018; Nunes et al., 2018, 2020; De Freitas et al., 2019; Jiménez-Reyes et al., 2019; Nikolaidis et al., 2019; Sekulic et al., 2019; Teixeira et al., 2019). This is extremely important, since understanding the match position-specific demands and the physical requirements for elite futsal players is the foundation for planning an effective training program. With this is in mind, the objective of this review is to update and summarize the current state of literature on the match-play demands and physical, physiological, and neuromuscular characteristics of elite futsal players and to present the differences between competition levels. To the best of the authors' knowledge, this is the first systematic review to simultaneously characterize futsal match-play demands through different approaches (i.e., time-motion analysis and wearable technology external load data, and physiological, neuromuscular, and biochemical responses) and describe the players' physical attributes.

## Methods

### Study Design

The present study is a systematic review focused on the match-play demands and players' characteristics (i.e., anthropometrics, physiological, and neuromuscular) at different levels of competition in futsal. The review was performed in accordance with the Preferred Reporting Items for Systematic Reviews and Meta-Analyses (PRISMA) statement (Moher et al., 2010) and did not require Institutional Review Board approval.

### Search Strategy

A systematic search was carried out in PubMed, Web of Science, and SportDiscus, all high-quality databases that assure a strong bibliographic support. The search strategy considered all the related articles published until July 25th, 2020. To ensure that all studies related to this topic were identified, a broad and general search was conducted by using solely the following keywords in the search strategy: (“futsal” OR “indoor soccer” OR “five-a-side soccer”). All titles and abstracts from the search were cross-referenced to identify duplicates and any missing studies and then screened for a subsequent full-text review. The search was performed independently by two authors (KS, EM-C), and any disagreement was resolved by a third party (TF).

### Inclusion and Exclusion Criteria

The review included cross-sectional and longitudinal studies published in English considering professional futsal players. The studies were included if they comprised (1) elite male futsal players; (2) sub-elite players, but only when compared to superior competition levels; (3) players ≥20 years old; and (4) variables related to the physical and physiological match-play demands and player characteristics (i.e., anthropometrics, physiological, or neuromuscular) were reported. Importantly, in the context of the current review, players were classified as elite if they competed in the National Team or 1st Division of their respective countries or in the 2nd Division of Spain, Portugal, Italy, or Russia. All the players that did not meet this standard were considered to be sub-elite.

Studies were excluded if (1) participants were ≤ 19 years old; (2) were female; (3) only sub-elite/state-level players participated in the study; (4) the division in which players competed was not detailed in the study (e.g., the players were referred to as “elite” but the article did not clearly mention that players competed in 1st Division); (5) non-English language; (6) the methodological quality assessment score was ≤ 8; and (7) the study consisted on a review or a conference paper.

### Study Selection

The initial search was conducted by two researchers (KS, EM-C). After the removal of duplicates, an intensive review of all the titles and abstracts obtained was completed and the ones not related to the review's topic were discarded. Following the systematic screening process, the full version of the remaining articles was read. All studies not meeting the inclusion criteria were then excluded.

### Data Extraction

Two reviewers (KS, EM-C) extracted the following data from the included studies: number and competitive level of the participants; match-play time-motion and physiological data; players' physiological and neuromuscular characteristics, the tests performed, measurement tools used, and outcome units. As the aim of the present review was not to investigate or determine the effects of different training programs on futsal players' physical qualities, in the studies in which interventions were used, the baseline values (i.e., pre-intervention) were extracted and reported in the respective tables in the Results section. In case the manuscript did not present numerical description of the data, the software GetData Graph Digitizer 2.26 (free software downloaded from http://getdata-graph-digitizer.com) was used to extract the outcome values from the articles' figures or graphs.

### Methodological Quality Assessment

The methodological quality of the included studies was evaluated separately by two researchers (KS, EM-C) using the modified scale of Downs and Black (Downs and Black, 1998) (Supplementary Material). Disputes were resolved by a third party (TTF). Of the 27 criteria, 12 were applied according to the study's design, as observed in similar research previously published (Cummins et al., 2013; Whitehead et al., 2018).

## Results

### Search Results

**Figure 1:**
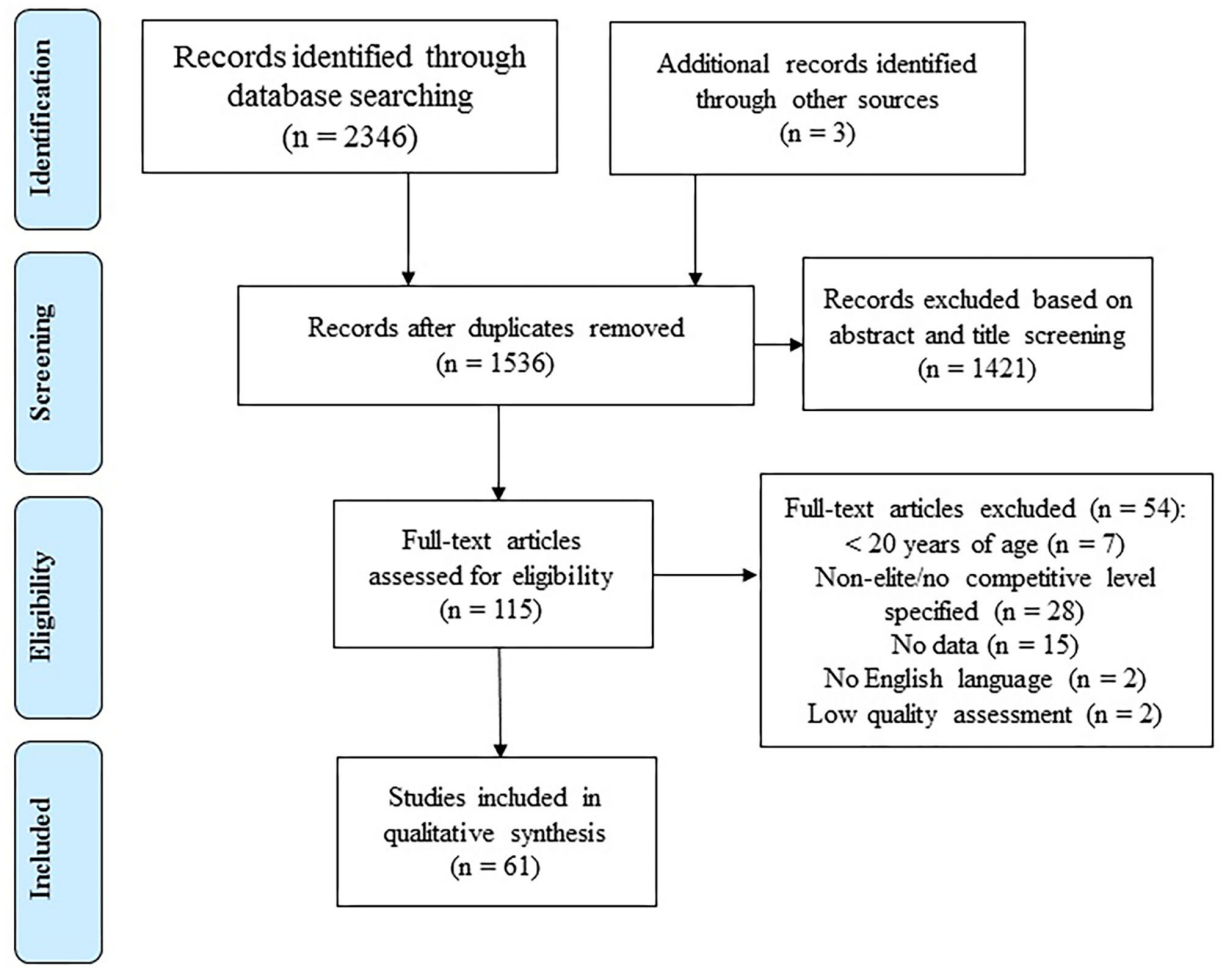
Flow diagram.

Figure 1 depicts the PRISMA flow diagram of the search and selection process of the studies. The initial databases yielded 2,346 citations, and 3 additional records were added through other sources. After duplicate removal, 1,536 articles remained. Upon title and abstract screening, 115 were left for the full-text review. Of the 115 articles reviewed, 61 met the criteria for the systematic review. Of the 61 the studies, 12 included time-motion analysis and match external load data, eight reported physiological responses to competition, four presented neuromuscular responses, and three considered biochemical responses. Regarding players' characteristics, 10 studies included anthropometric outcomes, 33 detailed physiological variables, and 21 investigated neuromuscular capabilities. Most of the studies were included in more than one section of the manuscript.

### Match Demands

#### Time-Motion Analysis and External Match Load

Time-motion analysis and external load tracking is frequently used within team-sports to monitor and describe players' activity patterns and movements during competition (Mohr et al., 2005; Burgess et al., 2006; Ben Abdelkrim et al., 2007). In this context, total distance covered, average speed, the number and distance of sprints, accelerations (ACC), and decelerations (DEC) are the most commonly reported variables as they help describe match and players' positional demands (Bangsbo, 1994).

In futsal, several studies have used video analysis and computer-based tracking systems but only two studies have used wearable technology (GPS or accelerometer) to analyze match-play demands (Table 1) (Dogramaci and Watsford, 2006; Barbero-Alvarez et al., 2008; Castagna et al., 2009; Dogramaci et al., 2011; Makaje et al., 2012; Bueno et al., 2014; Caetano et al., 2015; Milioni et al., 2016; Milanez et al., 2020; Ohmuro et al., 2020; Ribeiro et al., 2020; Yiannaki et al., 2020). Most recently, Ribeiro et al. (2020) analyzed a series of kinematic, mechanical, and metabolic variables during match-play using GPS wearable devices (WIMU PRO™, Realtrack Systems, Almeria, Spain) and found that the total distance covered was 3,749 ± 1,123 m and sprinting (≥18.0 km·h^−1^) distance corresponded to 135 ± 54 m. Moreover, the authors reported that futsal players performed a great number of high-intensity ACC (87 ± 49) and DEC (80 ± 32), when compared to the total number of jumps (9 ± 4). Utilizing a different approach (i.e., time-motion analysis), Castagna et al. (2009) had previously investigated game activities and showed that high-intensity running (≥15.5 km·h^−1^) and sprinting (≥18.3 km·h^−1^) accounted for 12 and 5% of the whole match duration, respectively. Furthermore, players performed a sprint every 79 s with an average distance of 10.5 m, a duration of 1.95 s, and a recovery between sprints of <40 s (Castagna et al., 2009). According to Barbero-Alvarez et al. (2008), who investigated the activity profile and match demands of an official futsal match using a video-based system (AV Master 98—Fast Multimedia), the distance covered during a match was 4,313 ± 2,139 m and the mean distance covered per minute of play was 117.3 ± 11.6 m. Moreover, the mean distances in walking (0.37–3.6 km·h^−1^) and jogging (3.7–10.8 km·h^−1^) were 397 m and 1,792 m, respectively, which represent 9 and 40% of the total mean distance covered. The mean distance in medium intensity activity (10.9–18 km·h^−1^) was 1,232 m (i.e., 28.5% of total match), high-intensity running (18.1–25 km·h^−1^) 571 m (13.7%), and sprinting (≥25.1 km·h^−1^) 348 m (8.9%) (Barbero-Alvarez et al., 2008). In a different study, professional futsal players tracked over five matches (i.e., using DVideo software automatic tracking system) (Bueno et al., 2014) were reported to cover a total distance of 3,133 m (2,248 interquartile ranges [IQRs]) for the whole match and a total relative distance of 97.9 ± 16.2 during the 1st half and 90.3 ± 12.0 m·min^−1^ during the 2nd. These values were somehow lower than the ones found by Castagna et al. (2009), which could be explained by the fact that, in the latter study, players completed a simulated futsal match comprising four sets of 10 min, with a 5 min rest (i.e., different from an official game). The dissimilarities in match characteristics may have allowed players to maintain higher levels of activity due to the shorter working period.

**Table 1:** Summary of time-motion analysis and physiological responses.

**Study**	**Participants (n^**°**^)**	**VO_**2max**_**	**Heart rate**	**Blood lactate (mmol·L^**−1**^)**	**Medium-intensity running**	**High-intensity running**	**Sprinting**	**Distance covered (m)**
Barbero-Alvarez et al. (2008)	10	NR	174 ± 7 b·min^−1^ 90% ± 2^d^	NR	1232 m 28% ± 2.2^c^	571 m 13.7% ± 2.0^c^	348 m 8.9% ± 3.4^c^	4313 ± 2138
Bekris et al. (2020)	21	NR	93% ± 2.5^d^	1^st^ half: 14.86 ± 4.91 2^nd^ half: 15.00 ± 4.67	NR	NR	NR	NR
Bueno et al. (2014)	93	NR	NR	NR	1^st^ half: 16.4% (IQRs: 3.4)^c^ 2^nd^ half: 15.4% (IQRs: 3.4)^c^	1^st^ half: 8.0% (IQRs: 2.4)^c^ 2^nd^ half: 7.5% (IQRs: 2.0)^c^	1^st^ half: 7.6% (IQRs: 4.3)^c^ 2^nd^ half: 7.2% (IQRs: 2.7)^c^	3133 (IQRs: 2248)
Caetano et al. (2015)	97	NR	NR	NR	NR	NR	26 ± 13.3 SP	NR
Castagna et al. (2009)	8	76% (95% CI: 59–92)^a^ 48.6 (95% CI: 40.1–57.1)^b^ ml·kg^−1^·min^−1^	90% (95% CI: 84–96)^a^	5.3 (95% CI: 1.1–10.4)	NR	12% (95% CI: 3.8–12.9)^c^	5% (95% CI: 1–11.0)^c^	NR
Charlot et al. (2016)	10	NR	168 ± 8.6 b·min^−1^ 83.2% ± 2.3^d^	NR	NR	NR	NR	NR
Dogramaci and Watsford (2006)	8	NR	NR	NR	1521 ± 558 m	1105 ± 384 m	106 ± 59.9 m	4283 ± 808
Dogramaci et al. (2011)	8	NR	NR	NR	999 ± 333 m	NR	106 ± 56	4277 ± 1030
Makaje et al. (2012)	15	OF: 77.9% ± 9.0^a^ 43.7 ± 5.8^b^ ml·kg^−1^·min^−1^ GL: 63.2% ± 8.9^a^ 31.5 ± 4.7^b^ ml·kg^−1^·min^−1^	OF: 175 ± 12^d^ b·min^−1^ 89.8% ± 5.8^d^ GL: 147 ± 7^d^ b·min^−1^ 73.7% ± 5.1^d^	OF: 5.5 ± 1.4 GL: 4.2 ± 1.3	OF: 1050 ± 355 m GL: 196 ± 130 m	OF: 636 ± 248 m GL: 127 ± 85 m	OF: 422 ± 186 m GL: 110 ± 57 m	OF: 5087 ± 1104 GL: 2043 ± 702
Milanez et al. (2020)	85	NR	NR	NR	NR	NR	NR	3046 ± 1485
Milioni et al. (2016)	10	NR	1^st^ half: 168.4 ± 12.4 b·min^−1^ 2^nd^ half: 166.4 ± 12.5 b·min^−1^	1^st^ half: 4.8 ± 2.3 2^nd^ half: 4.2 ± 2.2	NR	NR	1^st^ half: 49.5 ± 14.5 SP 2^nd^ half: 45.5 ± 9.1 SP	1^st^ half: 1986.6 ± 74.4 2^nd^ half: 1856 ± 127.7
Ohmuro et al. (2020)	79	NR	NR	NR	20% ± 2^c^	11.3% ± 1.4^c^	12% ± 3.1^c^	4151 ± 942^c^
Ribeiro et al. (2020)	28	NR	NR	NR	1321.5 ± 479.8 m	675.3 ± 298.1 m	134.9 ± 54.1 m	3749 ± 1123
Rodrigues et al. (2011)	14	79.2% ± 9.0^a^	86.4% ± 3.8^d^ 199 ± 8.5 b·min^−1^	NR	NR	NR	NR	NR
Yiannaki et al. (2020)	16	NR	87.7% ± 4.4^d^ 164.8 ± 22.3 b·min^−1^	NR	NR	NR	NR	NR

*Values expressed as mean ± SD*.

a *Mean game values with respect to maximal treadmill test values*.

b *Mean game values of VO_2_*.

c *Percentage of total playing time*.

d *Mean game values as percentage of maximum heart rate*.

*b·min^−1^, beats per minute; CI, confidence interval; GL, goalkeepers; IQRs, interquartile ranges; m, meters; n°, number; NR, not reported; OF, outfield player; SP, number of sprints; VO_2max_. maximum oxygen uptake*.

Examining the number of sprints performed during futsal competition, Caetano et al. (2015) observed, using a video automatic tracking system, that players execute 26 ± 13.3 sprints throughout the match. A more thorough analysis of the sprint demands noted that the most frequent repeated sprint actions comprised two consecutive sprints and a recovery of ~15 s. However, sequences of three and four sprints and rest intervals of 30, 45, or 60 s have also been reported. Considering playing position, there were no differences in sprint distance covered, peak velocity, initial velocity, recovery time between consecutive sprints, and number of sprints per min (Caetano et al., 2015). This could be explained not only by the tactical and technical characteristics of the sport (that make players more flexible to changing or rotating positions) but also by the unlimited number of substitutions or the possibility of playing with a “fly goalkeeper” during match-play.

Comparing the 1st and 2nd halves, a study with Brazilian elite players (Bueno et al., 2014) found that the percentage of distance covered standing and walking was higher in the 2nd half. Conversely, the distance covered at medium and high velocity and sprinting decreased significantly when compared to the 1st half (Bueno et al., 2014), which supported previous research (Barbero-Alvarez et al., 2008; Ribeiro et al., 2020). A related study (Milioni et al., 2016) confirmed that the total distance (1st half: 1,986 ± 74.4 m; 2nd half: 1,856 ± 129.7 m) and the distance covered by minute (1st half: 103.2 ± 4.4 m·min^−1^; 2nd half: 96.4 ± 7.5 m·min^−1^) decreased significantly from the 1st to the 2nd half but found no meaningful differences regarding the number of sprints or total sprinting time. Despite these inconsistencies, it appears that intensity tends to decrease as the match approaches the final minutes, which may be due not only to increased fatigue levels but also to tactical decisions (e.g., longer possessions or the utilization of a “fly goalkeeper”) that “slow down” the game.

In an investigation analyzing international- and national-level futsal competition, Dogramaci et al. (2011) reported that elite teams covered a 42% greater total distance than sub-elite teams did (4,277 ± 1,030 m vs. 3,011 ± 999 m, respectively). Moreover, the former traveled a 58% greater jogging distance and covered a 93% higher distance while moving sideways or backward and completed a higher total number of activities (i.e., 468 ± 77 for elite; 306 ± 81 for sub-elite) (Dogramaci et al., 2011). Upon review of the included studies, it appears that elite futsal players perform more high-energy metabolic and mechanical activities during competition with shorter recovery times. Match-related fatigue may influence high-intensity efforts and sprinting time from the 1st to 2nd half. From an applied perspective, knowing the match demands, understanding the differences in performance between the two halves and between professional and semi-professional athletes could be helpful for strength and conditioning coaches and sport scientists. These data may assist in developing more adequate match-action-specific training strategies, thus enhancing performance and potentially reducing the risk of injury. Interestingly, only two studies (Ribeiro et al., 2020; Yiannaki et al., 2020) used wearable technology (i.e., GPS or accelerometry) during the games, highlighting the need for further research regarding the description of the external loads experienced by players during official competition.

#### Physiological Responses

Due to the frequent intermittent high-intensity actions that occur in most team-sports, researchers have long been interested in understanding the physiological stress imposed during the match by analyzing variables such as heart rate (HR), oxygen uptake (VO_2_), or blood lactate concentration ([La]) (Spencer et al., 2005; Impellizzeri et al., 2006; Ostojic et al., 2006). Particularly in futsal, eight studies (Barbero-Alvarez et al., 2008; Castagna et al., 2009; Rodrigues et al., 2011; Makaje et al., 2012; Charlot et al., 2016; Milioni et al., 2016; Bekris et al., 2020; Yiannaki et al., 2020) have investigated the physiological responses during a match (Table 1).

Barbero-Alvarez et al. (2008) monitored the HR (Polar Vantage NV) of 10 players during four competitive futsal matches. The HR_mean_ was 174 ± 7 b·min^−1^ (range: 164–181), which represented 90 ± 2% (range 86–93) of HR_max_. With HR being classified based on the percentage of time spent in different zones, players spent 0.3, 16, and 83% at intensities ≤ 65, 85–65, and ≥85% of HR_max_, respectively. Other data from official matches, however, displayed slightly lower HR values (86.4 ± 3.8% HR_max_) (Rodrigues et al., 2011). Comparing the two halves, different outcomes have been reported in the literature. On the one hand, a significant decrease in the percentage of time spent at an intensity ≥85% of HR_max_ was identified from the 1st to 2nd half (Barbero-Alvarez et al., 2008). On the other, no meaningful differences were found on HR_max_ (1st half: 186.9 ± 9.2 b·min^−1^; 2nd half: 185.7 ± 10.0 b·min^−1^) and HR_mean_ (1st half: 168.4 ± 12.4 b·min^−1^; 2nd half: 166.4 ± 12.5 b·min^−1^) (Milioni et al., 2016). According to Castagna et al. (2009), the mean HR_max_ achieved during a simulated futsal match corresponded to 90% of the maximal treadmill test values, with peak values reaching 98%. Based on these results, it appears that HR_max_ values during official competition are lower than the ones achieved in a simulated match (i.e., 4 × 10 min, with a 5 min intermission); however, more research is needed to clarify the differences between the 1st and 2nd halves.

Regarding VO_2_, a study reported that the mean game values (measured with a portable gas analyzer) were 48.6 ml·kg^−1^·min^−1^ (95% confidence intervals [95% CI]: 40.1–57.1 ml·kg^−1^·min^−1^) and that players spent 46% of the playing time (during a simulated match) at intensities higher than 80% of VO_2max_ (Castagna et al., 2009). Moreover, the mean and peak values achieved during the modified game corresponded to 76 and 99%, respectively, of the VO_2max_ obtained in a maximal treadmill test (Castagna et al., 2009). When it comes to official competition data, an average intensity of 79.2 ± 9.0% of VO_2max_ was achieved in terms of oxygen consumption (Rodrigues et al., 2011). Concerning the accumulation of lactate ([La]), a [La]_mean_ value of 5.3 (95% CI: 1.1–10.4) mmol·L^−1^ was reported after the previously mentioned simulated match investigated by Castagna et al. (2009). Interestingly, and following the same pattern observed in the other variables (i.e., HR and VO_2max_), this value was higher than official games, in which [La]_mean_ (analyzed by an electrochemical lactimeter YSI 1500) of 4.8 ± 2.3 mmol·L^−1^ (1st half) and 4.2 ± 2.2 mmol·L^−1^ (2nd half) were found (Milioni et al., 2016). Conversely, Bekris et al. (2020), using a portable blood analyzer, displayed higher values of [La]_mean_ (1st half: 14.9 ± 4.9 and 2nd half: 15.0 ± 4.7) as the assessment was performed throughout the match, when the player was taken out.

The knowledge about the physiological demands of futsal is of paramount importance since it offers information concerning the stress imposed upon the players during competition. The average intensity of effort during the matches is high (mainly ≥85% of HR_max_) with an important decrement of high-intensity efforts between the two halves.

#### Neuromuscular Responses

High-intensity efforts (e.g., sprinting, jumping, and changes of direction [COD]) play a significant role in team-sports. Several studies indicate that stronger and more powerful players (i.e., with better-developed neuromuscular capabilities) of different sports are prone to accelerate faster, jump higher, and change direction more rapidly (Newton et al., 2006; Loturco et al., 2016b; Freitas et al., 2019). Moreover, it has been shown that sport-specific activities such as kicking or tackling are also influenced by the ability of an athlete to generate greater levels of force and power (Marques et al., 2007; Loturco et al., 2016a). With this in mind, four studies (Caetano et al., 2015; Milioni et al., 2016; Milanez et al., 2020; Ribeiro et al., 2020) investigated the neuromuscular outcomes during and after a futsal match.

Of note, apart from the increases in sprint time from the 1st to 2nd half discussed above, important alterations in neuromuscular function have been identified after a futsal match (Caetano et al., 2015; Ribeiro et al., 2020). Particularly, decrements in peak force and voluntary activation (i.e., manifestations of fatigue) were present following match-play; moreover, these were significantly associated with a reduction in running actions (i.e., repeated high-intensity efforts and sprints) (Milioni et al., 2016). Nevertheless, future studies are necessary to better elucidate the mechanisms (i.e., if peripheral or central in origin) impairing performance and the time-course of recovery (i.e., when do values get back to pre-competition levels) following a futsal match. Therefore, coaches and strength and conditioning specialists are advised to closely monitor the training and competition load and promote post-match recovery strategies to minimize injury risk and to potentially maintain players' peak neuromuscular performance throughout the season and during match-congested periods.

#### Biochemical Responses

To better understand the actual futsal match-play demands, and following a more holistic approach to the study of the stress imposed by competition, some researchers have investigated different biochemical markers post-game. Particularly, three studies (Moreira et al., 2011; de Moura et al., 2013; Bekris et al., 2020) have focused on this topic. A biomarker associated with responses to exercise is the salivary immunoglobulin A (SlgA), and when decreased, its concentration may be a good marker of excessive training (Petersen and Pedersen, 2005). Moreira et al. (2011) collected unstimulated saliva samples to investigate the SIgA responses in professional futsal players and observed a decline in absolute concentration, secretion rate, and saliva flow following a futsal match, which proposes a general risk for respiratory tract infection incidence. Hence, according to the authors' recommendations, actions should be held to minimize contact with virus or reduce training load under such conditions. Bekris et al. (2020) examined the biochemical and metabolic responses as well as the muscle damage induced by futsal competition and identified increased creatine kinase (CK) levels and a reduced testosterone/cortisol ratio after the game from blood samples collected from the forearm vein.

As it could be expected, given that different positions have different demands and characteristics (Baroni and Leal Junior, 2010; Ramos-Campo et al., 2014), dissimilar stress levels occur in the biochemical and immune systems. Goalkeepers have been reported to have a significantly higher lactate dehydrogenase concentration and IL-6 when compared to on-court players after the match; however, no differences in serum CK were obtained among positions (de Moura et al., 2013). In practical terms, results from the literature suggest that futsal competition promotes a decrease of plasma SlgA, increased muscle soreness, CK levels at post and post 24 h, and different stress responses among positions. These findings should be considered by coaches, strength and conditioning professionals, and nutritionists in order to maximize athletes' performance. Useful strategies may be the utilization of different techniques to avoid overreaching in futsal players; for instance, antioxidant supplement, omega-3 fatty acid, and anti-inflammatory drug intake, as well as reducing the training load.

### Player Characteristics

#### Anthropometrics

Anthropometric characteristics (i.e., height, body mass, and body composition) are important components of physical fitness as it is well-accepted that, for example, excessive body fat can potentially impair performance in team-sports (Vila Suárez et al., 2008). Conversely, a greater percentage of muscle skeletal mass tends to increase sport performance as it contributes to energy production during high-intensity activities and enhances athletes' force production capabilities (Vila Suárez et al., 2008). In this context, several studies have investigated the anthropometric characteristics of futsal players with the database search yielding 10 articles (Baroni and Leal Junior, 2010; Gomes et al., 2011; Jovanovic et al., 2011; Garrido-Chamorro et al., 2012; de Moura et al., 2013; Pedro et al., 2013; Ramos-Campo et al., 2014; Galy et al., 2015; Nikolaidis et al., 2019; López-Fernández et al., 2020). In general, elite futsal players have been reported to weigh, on average, ~70 kg, to measure ~1.76 m of height and to display ~15% of body fat (Jovanovic et al., 2011; Garrido-Chamorro et al., 2012).

Investigations comparing elite players with their sub-elite counterparts found no significant differences in anthropometric characteristics (Pedro et al., 2013; López-Fernández et al., 2020). For example, López-Fernández et al. (2020) found similar fat mass between elite and sub-elite players. However, elite players demonstrated higher lean mass in the dominant and non-dominant legs when compared to lower-level players; moreover, the latter showed higher bilateral asymmetry in fat mass percentage. No meaningful differences were found between professional and semi-professional players in a sample of Brazilian futsal players (Pedro et al., 2013). Therefore, it is still unknown to what extent height and body mass may be adequate variables to discriminate athletes from different competition levels.

Regarding playing position, research indicates significant differences on anthropometric characteristics (Baroni and Leal Junior, 2010; Ramos-Campo et al., 2014). In a study comparing body fat percentage among positions, pivots presented the highest value, followed by goalkeepers, backs, and, lastly, wingers (Ramos-Campo et al., 2014). In contrast, a different investigation (de Moura et al., 2013) found that goalkeepers were slightly taller and heavier and had a higher percentage of body fat (1.78 ± 3.2 cm, 74 ± 2.5 kg, 13 ± 2%, respectively) than defenders (1.74 ± 1 cm, 69 ± 2 kg, 10 ± 2%), wingers (1.69 ± 3 cm, 68 ± 2 kg, 11 ± 2%), and pivots (1.73 ± 2 cm, 71 ± 2 kg, 10 ± 2%). Similar results were found by Baroni and Leal Junior (2010), who indicated that the 22 goalkeepers comprised in the study's sample were significantly heavier and taller than their 164 on-court counterparts. The lack of significant differences in body fat among on-court players could be explained by the fact that, in futsal, playing positions are highly variable during the game because of the tactical behaviors that require players to perform multiple positional demands in order to adapt to the team's tactical system. It should be highlighted, however, that it is not clear whether the higher body mass reported for goalkeepers consists of fat or muscle mass. Given the paucity of data and lack of clear reporting, further research is required to better clarify the positional differences in anthropometric characteristics of futsal players.

In summary, according to the literature, futsal players display a low percentage of fat, which seems to be commonplace among players from different playing on-court positions and different competitive levels. This information may be important to adjust training programs and should be considered on young talent-detection practices.

#### Physiological Characteristics

The aerobic energy system has a crucial role in futsal match-play since it is well-established that this system improves recovery after high-intensity exercise (Helgerud et al., 2001; Tomlin and Wenger, 2001). Futsal players perform around 4 km in a match, with frequent bouts of repeated sprints, ACC, and DEC with short recovery times, which supports the importance of a well-developed aerobic energy system (Barbero-Alvarez et al., 2008; Ribeiro et al., 2020). In addition, as reported above, players achieve mean and peak VO_2_ values during competition which correspond to their 76 and 99% of VO_2max_, respectively. Upon review, 31 studies (Barbero-Alvarez et al., 2009; Gorostiaga et al., 2009; Baroni and Leal Junior, 2010; Castagna and Barbero-Alvarez, 2010; Milanez et al., 2011; Makaje et al., 2012; Boullosa et al., 2013; Oliveira et al., 2013; Pedro et al., 2013; Cuadrado-Peñafiel et al., 2014; Miloski et al., 2014; Soares-Caldeira et al., 2014; De Freitas et al., 2015, 2019; Galy et al., 2015; Garcia-Tabar et al., 2015; Charlot et al., 2016; Floriano et al., 2016; Nakamura et al., 2016, 2018; Naser and Ali, 2016; Barbieri et al., 2017; Barcelos et al., 2017; Valladares-Rodriguez et al., 2017; Nogueira et al., 2018; Zarebska et al., 2018, 2019; Farhani et al., 2019; Nikolaidis et al., 2019; Teixeira et al., 2019; Wlodarczyk et al., 2019, 2020; Bekris et al., 2020) have looked at the physiological characteristics of elite futsal players (Table 2).

**Table 2:** Summary of physiological characteristics.

**Study**	**Participants (n^**°**^)**	**Level**	**Test**	**Test-specific outcome**	**VO_2max_ (ml·kg^−1^·min^−1^)**	**HR_max_ (b·min^−1^)**	**VT_2_ (ml·kg^−1^·min^−1^)**	**Blood lactate (mmol·L^−1^)**
Barbero-Alvarez et al. (2009)	11	Elite	TM	N/A	62.9 ± 5.3	191 ± 8	44.4 ± 4.6	12 ± 2.5
	13	Sub-Elite			55.2 ± 5.7	198 ± 13	39.1 ± 4.0	7.8 ± 1.6
Barbieri et al. (2017)	16	Elite	Futsal_circuit	N/A	NR	198.5 ± 8	NR	7.87 ± 0.7
Barcelos et al. (2017)	8	Elite	Yo-Yo IR2	764.4 ± 206.7 m	55.7 ± 2.8	184.5 ± 12.2	NR	NR
Baroni and Leal Junior (2010)	186	Elite	TM	N/A	58 ± 6.37	190.14 ± 8.42	51.25 ± 5.84	NR
Bekris et al. (2020)	21	Elite	Yo-Yo IR2	N/A	65.17	193.0 ± 8.39	NR	NR
Boullosa et al. (2013)	15	Elite	TM	N/A	57.25 ± 6.35	184 ± 5	NR	NR
	15		Yo-Yo IR1		NR	184 ± 15		8.17 ± 1.63
Castagna et al. (2009)	18	Elite	TM	N/A	65.1 ± 6.2	193 ± 2	45.2 ± 4.6	12.6 ± 2.3
	18		FIET		61.6 ± 4.6	191 ± 7	NR	12.6 ± 2.3
Charlot et al. (2016)	10	Elite	30-15 IFT	19.2 ± 0.6 km·h^−1^	53.1 ± 2.1	NR	NR	NR
Cuadrado-Peñafiel et al. (2014)	12	Elite	TM	N/A	62.95 ± 5.21	NR	NR	NR
De Freitas et al. (2015)	10	Elite	Yo-Yo IR1	1433 ± 344^a^ m	NR	198.4 ± 7.3^a^	NR	NR
De Freitas et al. (2019)	10	Elite	FIET	16.6 ± 0.3 km·h^−1^	NR	NR	NR	NR
Farhani et al. (2019)	18	Elite	FSPT	770.2 ± 34.6W 30.08 ± 1.77 s	NR	NR	NR	NR
	18	Sub-Elite		714.5 ± 34W 35.45 ± 1.59 s				
Floriano et al. (2016)	10	Elite	TM		49.06 ± 4.7	185 ± 11	NR	8.5 ± 2.1
	10		T-CAR		51.1 ± 4.7	189 ± 9		13.6 ± 2.4
Galy et al. (2015)	22	Elite	30-15 IFT	MEL-G: 18.71 ± 1.33 km·h^−1^ NMEL-G: 19.5 ± 0.6 km·h^−1^	MEL-G: 51.46 ± 3.2 NMEL-G: 52.74 ± 1.94	MEL-G: 193.56 ± 8.26 NMEL-G: 187.88 ± 12.68	NR	NR
Garcia-Tabar et al. (2015)	10	Elite	SRT	N/A	NR	192 ± 5	NR	10 km·h^−1^: 1.5 ± 0.4 12 km·h^−1^: 2.0 ± 0.6 14 km·h^−1^: 4.4 ± 1.2
Gorostiaga et al. (2009)	15	Elite	SRT	N/A	NR	13 km·h^−1^: 170.7 ± 8 14 km·h^−1^: 179.9 ± 6 15 km·h^−1^: 183.5 ± 5	NR	13 km·h^−1^: 4.2 ± 2.0 14 km·h^−1^: 6.4 ± 2.1 15 km·h^−1^: 6.2 ± 1.7
Makaje et al. (2012)	15 15	Elite Sub-Elite	TM/ Yo-Yo IR2	OF: 1558 ± 451 m GL: 900 ± 403 m OF: 1203 ± 660 m GL: 726 ± 316 m	OF: 60.4 ± 5.1 GL: 54.6 ± 5.7 OF: 57.2 ± 6.2 GL: 52.4 ± 3.5	NR	NR	NR
Milanez et al. (2011)	9	Elite	TM	N/A	59.6 ± 2.5	190.4 ± 6.4	42.2 ± 6.0	NR
Miloski et al. (2014)	12	Elite	Yo-Yo IR2	450 ± 95.2^a^ m	48.6 ± 3.9^a^	NR	NR	NR
Miloski et al. (2016)	12	Elite	MSRT	N/A	49.5 ± 3.5^a^	NR	NR	NR
Nakamura et al. (2016)	18	Elite	Yo-Yo IR1	1506.7 ± 287.1 m	NR	NR	NR	NR
Nakamura et al. (2018)	11	Elite	Yo-Yo IR1	1160 ± 472.61 m	NR	NR	NR	NR
Naser and Ali (2016)	8	Elite	FIET	1378 ± 228.1 m	NR	NR	NR	NR
	8	Sub-Elite		1018.1 ± 133.8 m				
Nikolaidis et al. (2019)	16	Elite	MSRT	10:20 ± 1:20 min	NR	192.2 ± 6.9	NR	NR
Nogueira et al. (2018)	15	Elite	Yo-Yo IR2	573.3 ± 193.4^a^ m	NR	NR	NR	NR
Oliveira et al. (2013)	15	Elite	Yo-Yo IR1	1244 ± 298^a^ m	NR	NR	NR	NR
Pedro et al. (2013)	9	Elite	TM		63.7 ± 4.1	189 ± 7	43.0 ± 4.1	NR
	11	Sub-Elite	TM	N/A	62.1 ± 4.4	204 ± 11	44.0 ± 3.8	
Soares-Caldeira et al. (2014)	6	Elite	Yo-Yo IR1	1280 ± 363^a^ m	NR	NR	NR	NR
	7	Elite		1291 ± 363^a^ m				
Teixeira et al. (2019)	28	Elite	FIET	15.89 ± 1 km·h^−1^	NR	NR	NR	NR
Valladares-Rodriguez et al. (2017)	13	Elite	30-15 IFT	20.2 ± 1.7 km·h^−1^	NR	189 ± 7	NR	NR
Wlodarczyk et al. (2019)	12	Elite	TM	N/A	57.53 ± 1.1	187.50 ± 10	38.45 ± 3.9	10.9 ± 1.2
Wlodarczyk et al. (2020)	12	Elite	TM	N/A	56.14 ± 3.1^a^	187.09 ± 10.3^a^	37.69 ± 4.2^a^	11 ± 2.1^a^
Zarebska et al. (2018)	14	Elite	TM	N/A	56.66 ± 2.62	187 ± 10	NR	11.4 ± 2
Zarebska et al. (2019)	11	Elite	TM	N/A	55.81 ± 3.94^a^	192.7 ± 7^a^	NR	11.6 ± 2.2^a^

*Values expressed as mean ± SD*.

a *Pre-intervention values*.

*b·min^−1^, beats per minute; FIET, futsal intermittent endurance test; FSPT, futsal special performance test; GL, goalkeeper; HR_max_, maximum heart rate; MSRT, multistage 20 m shuttle-run test; MEL-G, Melanesians group; NMEL-G, non-Melanesians group; n°, number; N/A, not applicable; NR, not reported; OF, outfield players; SRT, submaximal running test; T-CAR, Carminattis's test; TM, treadmill test; VO_2max_, maximum oxygen uptake; VT_2_, ventilator anaerobic threshold; Yo-Yo IR1, Yo-Yo intermittent recovery test level 1; Yo- Yo IR2, Yo- Yo intermittent recovery test level*.

Considering competition level, elite and sub-elite players display dissimilar aerobic capacities (Barbero-Alvarez et al., 2009; Makaje et al., 2012; Pedro et al., 2013; Naser and Ali, 2016; Farhani et al., 2019). For example, VO_2max_ values of 62.9 ± 5.3 ml·kg^−1^·min^−1^ were reported for elite vs. 55.2 ± 5.7 ml·kg^−1^·min^−1^ for sub-elite athletes. Moreover, elite players presented a VO_2_ at a ventilatory anaerobic threshold (VT_2_) of 44.4 ± 4.6 ml·kg^−1^·min^−1^ while sub-elite displayed 39.1 ± 4.0 ml·kg^−1^·min^−1^ (Barbero-Alvarez et al., 2009). Interestingly, a study found no significant differences in VO_2max_ and VO_2_ at VT_2_ (in an incremental test in which players used a gas analyzer) but reported that the speed at VT_2_ (S_vt2_) and speed at VO_2max_ (S_Vo2max_) were significantly higher in elite players when compared to their sub-elite counterparts (S_vt2_: 11.2 ± 1.0 vs. 10.0 ± 1.2 km·h^−1^; S_Vo2max_: 17.5 ± 0.9 vs. 15.2 ± 1.0 km·h^−1^) (Pedro et al., 2013). Similar results were found elsewhere, when comparing elite, sub-elite, and social futsal players, using the distance covered in the Futsal Intermittent Endurance Test (FIET) (Naser and Ali, 2016). Elite players covered a greater distance (1,378 ± 228 m) in relation to sub-elite (1,018 ± 133 m) and social players (781 ± 220 m) (Naser and Ali, 2016).

A detailed look at the published studies portrays that different kinds of tests have been used to assess aerobic performance in futsal (e.g., Yo-Yo IR1–IR2, FIET, 30-15 Intermittent Fitness Test, Futsal Circuit, and Carminatti's test) and that fitness field tests may be useful to evaluate the aerobic capacity on elite players (Castagna and Barbero-Alvarez, 2010; Boullosa et al., 2013; Garcia-Tabar et al., 2015; Floriano et al., 2016; Barbieri et al., 2017; Valladares-Rodriguez et al., 2017). For example, a study by Nakamura et al. (2016) showed that Brazilian elite players covered 1,500 ± 287 m in the Yo-Yo IR1 test whereas a sample of under-20 players completed only 1,264.0 ± 397.9 m. Thus, it appears that such type of protocols may differentiate athletes from different age categories. A practical way to apply these field tests is through their implementation as part of the training routine as they may be equally useful for training purposes and performance monitoring. Moreover, the tests are inexpensive and need little equipment and few resources and player motivation could be increased when tests are completed with the ball.

The present findings suggest that physiological capacities may help discriminate superior-level futsal players since elite competitors display slightly higher VO_2max_ and VT_2_ values and obtain superior scores in different field tests in comparison with their sub-elite counterparts. Moreover, on-court players have greater aerobic capacity when compared to goalkeepers. Strength and conditioning coaches and sport scientists should focus on designing training drills that favor the improvement of the aerobic capacity to prepare players to cope with the demands of match-play.

#### Neuromuscular Characteristics

##### Strength capability

Strength and power capabilities are key components in most team-sports. Several studies have presented that stronger and more powerful players of different modalities tend to be faster, have better COD ability, and jump higher (Wilson et al., 1993; Newton et al., 2006; Freitas et al., 2019). In this context, four studies (Cuadrado-Peñafiel et al., 2014; Vieira et al., 2016; De Lira et al., 2017; Nunes et al., 2018) investigated the strength capabilities of futsal players (Table 3). Utilizing an isokinetic dynamometer, different authors (Vieira et al., 2016; De Lira et al., 2017; Nunes et al., 2018) assessed elite futsal players' strength levels by reporting peak torque values of the quadriceps and hamstrings. De Lira et al. (2017) reported that peak torque values at 60°·s^−1^ of the dominant leg were 223.9 ± 33.4 N·m for the quadriceps and 128 ± 27.6 N·m for the hamstrings, while the non-dominant leg displayed values of 224 ± 35.8 N·m and 124.1 ± 20.1 N·m^−1^ for the knee extensors and flexors, respectively. The H/Q ratio was 0.58 ± 0.1. Interestingly, when the mixed H/Q ratio (i.e., hamstrings eccentric angular velocity of 30°·s^−1^ and quadriceps concentric velocity of 240°·s^−1^) was assessed in the preferred and non-preferred limbs of 40 players, significant contralateral differences were found on knee flexors' eccentric contractions and in the H/Q ratio in favor of the preferred limb (Nunes et al., 2018). Only one study assessed the one repetition-maximum (1RM) on the half-squat exercise in order to characterize futsal players' strength qualities (1RM: 94.73 ± 17.01 kg) (Cuadrado-Peñafiel et al., 2014).

**Table 3:** Summary of neuromuscular characteristics.

**Study**	**Participants (n^**°**^)**	**Level**	**Strength**		**Jump (cm)**	**Sprint**	**COD (^**°**^)**
Charlot et al. (2016)	10	Elite	NR		NR	5 m: 1.00 ± 0.07 s 10 m: 1.72 ± 0.07 s 15 m: 2.38 ± 0.05 s 30 m: 4.20 ± 0.11 s	NR
Cuadrado-Peñafiel et al. (2014)	12	Elite	1RM Squat: 94.73 ± 17.01 kg		CMJ: 35.9 ± 5.29	NR	NR
De Freitas et al. (2019)	10	Elite	NR		SJ: 34.6 ± 3.9^a^ CMJ: 36.6 ± 4.1^a^	15 m: 2.43 ± 0.12^a^ s	NR
De Lira et al. (2017)	30	Sub-Elite	60°·s^−1^ (N·m)	Ext Dom: 223.9 ± 33.4 Ext N-Dom: 224 ± 35.8 Flex Dom: 128 ± 27.6 Flex N-Dom: 124.1 ± 20.1	NR	NR	NR
Galy et al. (2015)	22	Elite	NR		MEL-G: CMJ: 50.44 ± 5.88 NMEL-G: CMJ: 45.16 ± 4.34	MEL-G: 5 m: 1.41 ± 0.11 s 10 m: 2.18 ± 0.12 s 15 m: 2.82 ± 0.15 s 30 m: 4.72 ± 0.17 s NMEL-G: 5 m: 1.35 ± 0.08 s 10 m: 2.13 ± 0.13 s 15 m: 2.84 ± 0.12 s 30 m: 4.80 ± 0.15 s	T-Test (90/180°): MEL-G: 10.47 ± 0.58 s NMEL-G: 11.01 ± 0.64 s
Gomes et al. (2011)	92	Elite	NR		SJ: 36.74 ± 4.28 37.42 ± 4.86 36.61 ± 5.28 CMJ: 38.88 ± 4 39.72± 5.08 38.48 ± 4.80	NR	NR
Gorostiaga et al. (2009)	15	Elite	NR		CMJ: 38.1 ± 4.1	5 m: 1.01 ± 0.02 s 15 m: 2.41 ± 0.08 s	NR
Jiménez-Reyes et al. (2019)	39	Elite	NR		NR	5 m: 1.36 ± 0.04 s 20 m: 3.36 ± 0.09 s	NR
	10	Sub-Elite				5 m: 1.40 ± 0.02 s 20 m: 3.46 ± 0.04 s	
Loturco et al. (2018)	63	Elite	NR		SJ: 37.82 ± 7.10 CMJ: 38.50 ± 4.88	5 m: 4.81 ± 0.25 m·s^−1^ 10 m: 5.68 ± 0.19 m·s^−1^ 20 m: 6.61 ± 0.22 m·s^−1^	Zig-zag (100°): 3.52 ± 0.11 m·s^−1^
Loturco et al. (2020)	62	Elite	NR		NR	5 m: 4.79 ± 0.22 m·s^−1^ 10 m: 5.67 ± 0.23 m·s^−1^ 20 m: 6.62 ± 0.25 m·s^−1^	Zig-zag (100°): 3.52 ± 0.16 m·s^−1^ COD-Def zig-zag (100°): 3.09 ± 0.25 m·s^−1^
Miloski et al. (2016)	12	Elite	NR		CMJ: 47.5 ± 5.5^a^	5 m: 1.10 ± 0.08^a^ s 20 m: 3.14 ± 0.11^a^ s	T-Test 90/180°: 9.24 ± 0.31^a^ s
Nakamura et al. (2016)	18	Elite	NR		SJ: 37.75 ± 3.93 CMJ: 39.22 ± 4.42	5 m: 1.05 ± 0.04 s 10 m: 1.78 ± 0.06 s 20 m: 3.05 ± 0.10 s	Zig-zag (100°): 5.71 ± 0.22 s
Naser and Ali (2016)	8	Elite	NR		CMJ: 52.1 ± 4.2	5 m: 1.00 ± 0.04 s 10 m: 1.75 ± 0.03 s 20 m: 2.99 ± 0.04 s	NR
	8	Sub-Elite			CMJ: 49.9 ± 3.9	5 m: 1.06 ± 0.02 s 10 m: 1.78 ± 0.01 s 20 m: 3.05 ± 0.04 s	
Nikolaidis et al. (2019)	16	Elite	NR		ABK: 38.9 ± 6.1	10 m: 1.85 ± 0.12 s 20 m: 3.18 ± 0.17 s	NR
Nogueira et al. (2018)	15	Elite	NR		SJ: 36.31 ± 4.08^a^ CMJ: 40.11 ± 4.73^a^ DJ: 38.33 ± 4.75^a^	NR	NR
Nunes et al. (2018)	40	Elite	60°·s^−1^ (N·m)	Ext Pref: 214.7 ± 49.6 Ext N-Pref: 216.5 ± 51.6 Flex Pref: 136.6 ± 31.7 Flex N-Pref: 135.8 ± 3	NR	NR	NR
			240°·s^−1^ (N·m)	Ext Pref: 178.1 ± 53.16 Ext N-Pref: 176.8 ± 52 Flex Pref: 124.3 ± 40.3 Flex N-Pref: 115.9 ± 38.1			
			30°·s^−1^ Ecc (N·m)	Ext Pref: 296 ± 75.7 Ext N-Pref: 277.2 ± 73 Flex Pref: 173.5 ± 35.8 Flex N-Pref: 162.9 ± 40.8			
			120°·s^−1^ Ecc (N·m)	Ext Pref: 299.3 ± 66.4 Ext N-Pref: 277.3 ± 66.1 Flex Pref: 185.7 ± 35.8 Flex N-Pref: 172.7 ± 58			
Nunes et al. (2020)	20	Elite	NR		SJ: 36.6 ± 3.2^a^ 35.7 ±3.6^a^ CMJ: 39.4 ± 3.4^a^ 38.6 ± 3.9	5 m: 1.07 ± 0.04^a^ s 1.06 ± 0.05^a^ s 10 m: 1.39 ± 0.04^a^ s 1.37 ± 0.05^a^ s 15 m: 2.52 ± 0.06^a^ s 2.52 ± 0.10^a^ s	NR
Sekulic et al. (2019)	12	Elite	NR		NR	10 m: 1.63 ± 0.07 s	CODS_DD (38°): 2.39 ± 0.19 s CODS_DND 38°): 2.57 ± 0.16 s CODS_TD 38°): 2.03 ± 0.11 s COD_TND 38°): 2.31 ± 0.12 s
	20	Sub-Elite				10 m: 1.69 ± 0.11 s	CODS_DD (38°): 2.57 ± 0.22 s CODS_DND 38°): 2.65 ± 0.15 s CODS_TD 38°): 2.08 ± 0.14 s COD_TND 38°): 2.23 ± 0.10 s
Soares-Caldeira et al. (2014)	6	Elite	NR		SJ: 33.13 ± 5.76 CMJ: 38.82 ± 6.39	NR	NR
	7	Elite			SJ: 34.47 ± 2.50 CMJ: 42.77 ± 2.78		
Teixeira et al. (2019)	28	Elite	NR		SJ: 34.42 ± 4.15^a^ CMJ: 35.37 ± 3.65^a^	5 m: 4.75 ± 0.46^a^ m·s^−1^ 15 m: 6.21 ± 0.37^a^ m·s^−1^	NR
Vieira et al. (2016)	17	Elite	60°·s^−1^ (N·m)	Ext Dom: 253.31 ± 33.81 Ext N-Dom: 244.83 ± 24.78	NR	NR	NR
			180°·s^−1^ (N·m)	Ext Dom: 184.04 ± 18.84 Ext N-Dom: 182.86 ± 20.17			
			300°·s^−1^ (N·m)	Ext Dom: 138.59 ± 17.27 Ext N-Dom: 142.33 ± 18.77			

*Values expressed as mean ± SD*.

a *Pre-intervention values*.

*ABK, Abalakov jump test; cm, centimeter; CMJ, countermovement jump test; COD, change of direction; COD-Def, change of direction Deficit; CODS_DD, change of direction dominant leg with ball; CODS_DND, change of direction of non-dominant leg with ball; CODS_TD, change of direction of dominant leg without ball; CODS_TND, change of direction of non-dominant leg without ball; Dom, dominant leg; Ecc, eccentric; Ext, extensor; Flex, flexor; HJ, horizontal jump test; L, left leg; MEL-G, Melanesians group; NMEL-G, non-Melanesians group; n°, number; N-Dom, non-dominant leg; N-Pref, non-preferred leg; NR, not reported; Pref, preferred leg; R, right leg; s, seconds*.

Considering the previous, more research is clearly needed to investigate the force production capabilities of futsal athletes, as the vast majority of research utilized isokinetic dynamometry. Accordingly, the dominant leg seems to be stronger (i.e., reach higher peak torque values) than the non-dominant leg. Based on this information, strength and conditioning specialists should be aware that unilateral strength testing may be necessary to allow preparing specialized and tailored training plans to maximize lower-body strength and attenuate the likelihood of injuries. However, given that isokinetic testing is extremely time-consuming, expensive, and not practical to use in real-world scenarios, other exercises (e.g., half-squat, split squat, hip-thrust, or deadlift, isometric tests) should be implemented when assessing lower-body strength.

##### Jumping ability

Data from futsal competition indicates that players perform multiple high-intensity efforts (i.e., jumping, sprinting, or COD) (Caetano et al., 2015; Ribeiro et al., 2020). For that reason, and considering that lower-body powerful actions are determinant during the match, several researchers have investigated power-related capacities of futsal players. Particularly, 14 studies (Gorostiaga et al., 2009; Gomes et al., 2011; Cuadrado-Peñafiel et al., 2014; Soares-Caldeira et al., 2014; Galy et al., 2015; Miloski et al., 2016; Nakamura et al., 2016; Naser and Ali, 2016; Loturco et al., 2018; Nogueira et al., 2018; De Freitas et al., 2019; Nikolaidis et al., 2019; Teixeira et al., 2019; Nunes et al., 2020) assessed elite futsal players' jumping ability (Table 3). For example, an investigation with 63 professional players reported jump heights (measured with a contact mat) of 37.8 cm in the squat jump and 38.5 cm in the countermovement jump (CMJ) as well as bar mean propulsive and peak power outputs of 9.2 and 20.4 W·kg^−1^, respectively (Loturco et al., 2018). Similar values for the CMJ were reported elsewhere (Gorostiaga et al., 2009).

Considering the different levels of futsal players, Naser and Ali, 2016 identified no significant differences in CMJ height between elite and sub-elite futsal players. Despite the need for players to execute vertical jump actions during futsal competition, it seems that these may be less determinant for performance when compared to other sports, such as soccer. Based on the studies that have assessed vertical jump height, it appears that elite futsal players do not display greater jumping ability than their sub-elite counterparts, potentially due to the limited influence of jumping ability in the game. However, it has been shown that the successful application of vertical ground reaction forces (i.e., as in vertical jumping) may play a significant role in multiple athletic actions (e.g., sprinting or COD) (Loturco et al., 2019). For this reason, strength and conditioning coaches are encouraged to include multiple bilateral and unilateral jumping tasks in their training programs to maximize lower-body power and, consequently, performance of elite futsal players.

##### Sprinting ability

Data from match demands demonstrates that futsal players perform ~26 sprints with an average duration of 2–4 s over 8–20 m (Caetano et al., 2015). Considering that, several authors (Gorostiaga et al., 2009; Galy et al., 2015; Charlot et al., 2016; Miloski et al., 2016; Nakamura et al., 2016; Naser and Ali, 2016; Loturco et al., 2018, 2020; De Freitas et al., 2019; Jiménez-Reyes et al., 2019; Nikolaidis et al., 2019; Sekulic et al., 2019; Teixeira et al., 2019; Nunes et al., 2020) investigated the sprint performance of futsal players (Table 3). Loturco et al. (2018) utilized photocells to examine sprint capabilities and found velocities (i.e., average velocity derived from time and distance) of 4.81 ± 0.25 m·s^−1^ (5 m), 5.68 ± 0.19 m·s^−1^ (10 m), and 6.61 ± 0.22 m·s^−1^ (20 m) in elite futsal players. Regarding acceleration ability (i.e., calculated as the rate of change of velocity with respect to time), the same study reported values of 4.64 ± 0.50 m·s^−2^ for 0–5 m, 1.22 ± 0.22 m·s^−2^ for 5–10 m, and 0.74 ± 0.09 m·s^−2^ for 10–20 m. Gorostiaga et al. (2009) assessed 5 and 15 m sprint times (not velocities) of 15 players (using photocell gates) and found values of 1.01 ± 0.02 and 2.41 ± 0.08 s, respectively.

Regarding competition level, a training approach based on the force–velocity profile found that 1st-Division futsal players sprinted 5 m in 1.36 ± 0.04 s and 20 m in 3.36 ± 0.09 s while 2nd-Division players demonstrated lower sprint performances (5 m: 1.40 ± 0.02 s; and 20 m: 3.46 ± 0.04 s) (Jiménez-Reyes et al., 2019). Along the same lines, other studies (Naser and Ali, 2016; Sekulic et al., 2019) observed that elite futsal players run faster over 5, 10, and 20 m than sub-elite or social players.

From the above information, it appears that elite players tend to display higher sprinting ability when compared to their sub-elite peers, although further research is necessary. Nevertheless, given that the majority of the published literature indicates that higher-level players tend to be faster, short sprints should be seen as an important training stimulus that may enhance the players' ability to succeed at superior competition levels, where match demands are greater.

##### Change of direction ability and agility

COD is one of most important efforts in futsal due to the rapid changes of activity during the match. COD relies on a series of anthropometric (e.g., height, leg length), physical (e.g., lower-body and trunk muscular strength, speed-power-related capabilities), and technical aspects (e.g., stride adjustments, foot placement) (Jeffreys, 2008; Pereira et al., 2018). In this context, six investigations (Galy et al., 2015; Miloski et al., 2016; Nakamura et al., 2016; Loturco et al., 2018, 2020; Sekulic et al., 2019) have performed an in-depth analysis of this paramount ability in futsal players (Table 3). In a study that examined COD performance on different sports, including futsal, players performed a zig-zag test consisting of four 5 m sections marked with cones set at 100° angles. The results found that futsal players obtained a COD velocity of 3.52 ± 0.11 m·s^−1^ (Loturco et al., 2018). When a complementary investigation from the same research group assessed the “COD deficit” (i.e., the difference in velocity between a linear sprint and a COD task of equivalent distance) for the first time in futsal, players from this modality were found to be more efficient than soccer players at changing direction but displayed COD deficits similar to other team-sports (i.e., rugby and handball players) (Loturco et al., 2020). Of note, a unique investigation (Sekulic et al., 2019) designed a “Y” -shaped pattern test to evaluate COD and agility in futsal with and without ball using a timing gate system. The COD and agility assessments without the ball requested participants to touch the ball and change direction; with ball, players had to dribble and conduct the ball during the execution of each test. In the COD test, participants had advanced knowledge of the task and knew which cone would light up. In contrast, the agility drill was not planned, and players needed to identify a stimulus and react accordingly. The results demonstrated that both tests were reliable after trials of submaximal intensity, with lower reliability of the non-dominant leg (Sekulic et al., 2019).

In summary, further investigations regarding COD ability are needed in futsal. Strength and conditioning coaches should implement COD training during tactical–technical sessions or develop ACC-DEC capabilities through the use of other training approaches (i.e., resisted sprints, horizontally oriented power exercises, or eccentric training) given the importance of COD maneuvers in futsal.

## Limitations

Some limitations should be addressed when considering the present research. Firstly, the number of studies assessing each variable is quite different, which means that the evidence level is dissimilar among variables. For example, there are more studies describing the match-play demands via time–motion analysis than describing the strength or COD capacities of futsal players. Secondly, the instruments, tests, or data collection procedures differed among studies, which precluded a direct comparison and interpretation of the data in some occasions. Further studies are still necessary to have a clearer picture of the futsal match-play demands, particularly, using new technologies (e.g., GPS or accelerometry-based). In addition, more research into the athletes' physical characteristics and performance outcomes (and how they fluctuate across a competitive season) would bring further understanding on the neuromuscular profile of futsal players.

## Conclusion

This systematic review provides useful information for strength and conditioning coaches and sport scientists regarding the match demands, anthropometric characteristics, and physical qualities of elite and sub-elite male futsal players. The results indicated that futsal is characterized by intermittent high-intensity activities with a great number of ACC, DEC, and sprints; short recovery times between them; and multiple COD actions during match-play. The abundance of these types of efforts produces important decrements in physiological and neuromuscular responses between the two halves and immediately following match-play. Moreover, biochemical responses appear to be affected up to 24 h after the match. Comparing competition level, differences were observed in match demands, with elite players covering higher distance, performing more high-intensity actions, and presenting lower standing time when compared to sub-elite players. An analysis of the anthropometric characteristics of futsal players showed low percentages of body fat with no differences between on-court players of different positions or level of competition. However, goalkeepers were found to present higher body fat. Regarding the physiological characteristics of futsal players, these display VO_2max_ values of around 62 ml·kg^−1^·min^−1^. Elite futsal players possess higher VO_2max_, when compared to their sub-elite counterparts. From the present review, it can be concluded that further investigation on the neuromuscular capabilities (i.e., strength, jumping, and COD) of futsal players is warranted. Still, it appears that elite futsal players present better sprinting abilities when compared to lower-level players but that jumping capacity seems not to differentiate between competition levels. Futsal players aiming to compete at the highest level should focus on developing maximal speed, lower-body power and strength, aerobic capacity, and lean muscle mass.

## Author Contributions

KS, TF, and PA designed this study. Research literature was conducted by KS and EM-C. KS drafted the manuscript. All authors revised the manuscript and approved the final version to be published.

## Conflict of Interest

The authors declare that the research was conducted in the absence of any commercial or financial relationships that could be construed as a potential conflict of interest.
