# Supplementary material for: Physical and Physiological Match-Play Demands and Player Characteristics in Futsal: A Systematic Review
Source: Front Psychol. 2020 Nov 6;11:569897. doi: 10.3389/fpsyg.2020.569897 (PMC7677190; doi:10.3389/fpsyg.2020.569897)
Supplement: Supplementary file 1 [file Data_Sheet_1.pdf]

Methodological Quality Assessment

Methodological Quality Assessment Downs and Black													
Study	Question Number												Total Score
	1	*2*	*3*	*4*	*6*	*7*	*10*	*11*	*12*	*16*	*18*	*20*	
Barbero-Alvarez et al. (2008)	1	1	1	1	1	1	1	0	0	1	1	1	10
Barbero-Alvarez et al. (2009)	1	1	1	1	1	1	1	0	0	1	1	1	10
Barbieri et al. (2017)	1	1	1	1	1	1	1	0	0	1	1	1	10
Barcelos et al. (2017)	1	1	1	1	1	1	0	0	0	1	1	1	9
Baroni and Leal Junior (2010)	1	1	1	1	1	1	1	0	0	1	1	1	10
Bekris et al. (2020)	1	1	1	1	1	1	1	0	0	1	1	1	10
Boullosa et al. (2013)	1	1	1	1	1	1	1	0	1	1	1	1	11
Bueno et al. (2014)	1	1	1	1	1	1	1	0	0	1	1	1	10
Caetano et al. (2015)	1	1	0	1	1	1	1	0	0	1	1	1	9
Castagna et al. (2009)	1	1	1	1	1	1	0	0	0	1	1	1	9
Castagna et al. (2010)	1	1	1	1	1	1	1	0	0	1	1	1	10
Charlot et al. (2016)	1	1	1	1	1	1	1	0	0	1	1	1	10
Cuadrado-Peñafiel et al. (2014)	1	1	1	1	1	1	0	0	0	1	1	1	9
De Freitas et al. (2015)	1	1	1	1	1	1	0	0	0	1	1	1	9
De Freitas et al. (2019)	1	1	1	1	1	1	1	0	0	1	1	1	10
De Lira et al. (2017)	1	1	1	1	1	1	1	0	0	1	1	1	10
de Moura et al. (2013)	1	1	1	1	1	1	0	0	0	1	1	1	9
Dogramaci and Watsford (2006)	1	1	1	1	1	1	0	0	0	1	1	1	9
Dogramaci et al. (2011)	1	1	1	1	1	1	1	0	0	1	1	1	10
Farhani et al. (2019)	1	1	1	1	1	1	1	0	0	1	1	1	10
Floriano et al. (2016)	1	1	1	1	1	1	1	0	0	1	1	1	10
Galy et al. (2015)	1	1	1	1	1	1	1	0	0	1	1	1	10
Garcia-Tabar et al. (2015)	1	1	1	1	1	1	1	0	0	1	1	1	10
Garrido-Chamorro et al. (2012)	1	1	1	1	1	1	0	0	0	1	1	1	9
Gomes et al. (2011)	1	1	1	1	1	1	1	0	0	1	1	1	10
Gorostiaga et al. (2009)	1	1	1	1	1	1	0	0	0	1	1	1	9
Jiménez-Reyes et al. (2019)	1	1	1	1	1	1	0	0	0	1	1	1	9
Jovanovic et al. (2011)	1	1	1	1	1	1	1	0	0	1	1	0	9
Lopéz-Fernández et al. (2020)	1	1	1	1	1	1	1	0	0	1	1	1	10
Loturco et al. (2018)	1	1	1	1	1	1	0	0	0	1	1	1	9
Loturco et al. (2020)	1	1	1	1	1	1	0	0	0	1	1	1	9
Makaje et al. (2012)	1	1	1	1	1	1	0	0	0	1	1	1	9
Milanez et al. (2011)	1	1	1	1	1	1	0	0	0	1	1	1	9
Milanez et al. (2020)	1	1	0	1	1	1	1	0	1	1	1	1	10
Milioni et al. (2016)	1	1	1	1	1	1	1	0	0	1	1	1	10
Miloski et al. (2014)	1	1	1	1	1	1	1	0	0	1	1	1	10

Miloski et al. (2016)	1	1	1	1	1	1	1	0	0	1	1	1	10
Moreira et al. (2011)	1	1	1	1	1	1	0	0	0	1	1	1	9
Nakamura et al. (2016)	1	1	1	1	1	1	1	0	0	1	1	1	10
Nakamura et al. (2018)	1	1	1	1	1	1	0	0	0	1	1	1	9
Naser and Ali (2016)	1	1	1	1	1	1	1	0	0	1	1	1	10
Nikolaidis et al. (2019)	1	1	1	1	1	1	1	0	0	1	1	1	10
Nogueira et al. (2018)	1	1	1	1	1	1	0	0	0	1	1	1	9
Nunes et al. (2018)	1	1	1	1	1	1	1	0	0	1	1	1	10
Nunes et al. (2020)	1	1	1	1	1	1	1	0	0	1	1	1	10
Ohmuro et al. (2020)	1	1	1	1	1	1	0	0	0	1	1	1	9
Oliveira et al. (2013)	1	1	1	1	1	1	1	0	0	1	1	1	10
Pedro et al. (2013)	1	1	1	1	1	1	1	0	0	1	1	1	10
Ramos-Campo et al. (2014)	1	1	1	1	1	1	0	0	0	1	1	1	9
Ribeiro et al. (2020)	1	1	0	1	1	1	1	0	0	1	1	1	9
Rodrigues et al. (2011)	1	1	1	1	1	1	0	0	0	1	1	1	9
Sekulic et al. (2019)	1	1	1	1	1	1	1	0	0	1	1	1	10
Soares-Caldeira et al. (2014)	1	1	1	1	1	1	1	0	0	1	1	1	10
Teixeira et al. (2019)	1	1	1	1	1	1	0	0	0	1	1	1	9
Valladares-Rodríguez et al. (2017)	1	1	1	1	1	1	0	0	0	1	1	1	9
Vieira et al. (2016)	1	1	1	1	1	1	0	0	1	1	1	1	10
Wlodarczyk et al. (2019)	1	1	1	1	1	1	1	0	1	1	1	1	11
Wlodarczyk et al. (2020)	1	1	1	1	1	1	1	0	1	1	1	1	11
Yiannaki et al. (2020)	1	1	1	1	1	1	0	0	0	1	1	1	9
Zarebska et al. (2018)	1	1	1	1	1	1	1	0	0	1	1	1	10
Zarebska et al. (2019)	1	1	1	1	1	1	1	0	0	1	1	1	10
